# Construction of an individualized clinical prognostic index based on ubiquitination-associated lncRNA in clear cell renal cell carcinoma patients

**DOI:** 10.1186/s12957-022-02618-x

**Published:** 2022-05-10

**Authors:** Kun Liu, Xuzhong Liu, Qing Sun, Zhiwang Tang, Gongcheng Wang, Zongyuan Xu

**Affiliations:** Department of Urology, Huai’an First People’s Hospital, Nanjing Medical University, Huai’an, 223300 China

**Keywords:** Clear cell renal cell carcinoma, Ubiquitination, Long noncoding RNA, Clinical-prognostic index, Bioinformatic analysis

## Abstract

**Background:**

ccRCC is considered as the main subtype of RCC, which accounted for sixth deadliest cancer worldwide. Recently, ubiquitination has been reported to be closely involved in the progression of tumore. The purpose of this study was to identify the ubiquitination-associated genes and co-expressed lncRNAs on the prognosis of clear cell renal cell carcinoma (ccRCC) patients.

**Methods and patients:**

We downloaded 530 cases and the corresponding transcriptome profiling from The Cancer Genome Atlas (TCGA) database. We distinguished mRNA and lncRNA expression data from the transcriptome profiling and then extracted the expression of mRNAs that regulate protein ubiquitination. We obtained lncRNAs associated with protein ubiquitination regulation from the lncRNA data by gene co-expression analysis. Cox regression analysis of survival time, survival status, and lncRNA expression level was carried out, and a prognostic index (PI) was constructed.

**Results:**

The PI was established based on 8 prognostic lncRNAs that regulate protein ubiquitination and distinguish the high-risk group patients from all patients. Multivariate analysis indicated that this PI was an individualized clinical prognostic factor for patients with ccRCC. Regarding clinical characteristics, a ubiquitination-associated clinical-prognostic index (UCPI), containing 8 ubiquitination-related lncRNAs and age, was established and tested with AUC of 0.80.

**Conclusion:**

We established a UCPI containing 8 lncRNAs related to protein ubiquitination. This UCPI may become an appropriate model to predict the prognosis in ccRCC patients and guide clinicians to adjust the follow-up regimen.

**Supplementary Information:**

The online version contains supplementary material available at 10.1186/s12957-022-02618-x.

## Introduction

Renal cell carcinoma (RCC) was the sixth deadliest cancer worldwide in 2018, accounting for approximately 4% of adult malignancies [1, 2]. According to the American Cancer Society, about 73,820 new cases of RCC were diagnosed by the end of 2019, and more than 14,770 deaths caused by RCC were reported in 2019 in the USA [3]. Considering pathological features, RCC has been classified as clear cell RCC (ccRCC), papillary RCC (pRCC), and chromophobe RCC (chRCC) [4]. Among them, ccRCC was considered the most prevalent subtype, accounting for about 75% of all RCCs [5]. Although surgery, radiotherapy, and chemotherapy have significantly improved over the past decades, ccRCC remains an aggressive cancer with a recurrence rate of up to 40% after initial treatment [6]. Therefore, there is an urgent need to explore and establish new biomarkers, or models, for ccRCC survival risk prediction to improve individualized treatment and long-term life quality.

Long noncoding RNAs (lncRNAs) are defined as transcripts with a length of less than 200 nucleotides and no protein-coding function [7]. Several studies explored and reported multiple functions of lncRNAs, such as transcription, translation, splicing, and cell differentiation regulation [8]. Furthermore, lncRNAs have been considered to play vital roles in cancer immunity and development and to encode small proteins in certain conditions [9, 10]. Increasing evidence supported the strong correlation of lncRNAs in the proliferation, metastasis, and prognosis of ccRCC, indicating the potential efficacy of lncRNAs to predict ccRCC patients’ risk [11, 12].

Ubiquitination is a crucial biological and physiological process in humans, dynamically modulating protein degradation marks through various mechanisms, including proteasome, cell activity regulation, and location switching [13, 14]. Since the isolation of the von Hippel-Lindau gene (VHL) in 1993, Bi-allelic *VHL* loss, either through mutation, hypermethylation, or chromosomal loss, has been widely reported in most ccRCC patients, indicating the pivotal role of VHL in the genetic pathogenesis of ccRCC [15]. Besides, *VHL* gene-dependent tumor suppression in ccRCC responded to the *VHL*-mediated ubiquitination process, enabling the possibility of ccRCC treatment through ubiquitination inhibition [16]. Moreover, ubiquitin-conjugating enzyme E2T has been reported to play a critical role in ccRCC progression and may be a potential therapeutic target for ccRCC [17]. Recently, ubiquitin-like modifier-activating enzyme 2, an important member of the SUMO modification system, was found to promote the cell growth of ccRCC [18]. Considering the ambiguous relationship between ubiquitination and prognosis of ccRCC, developing a lncRNA signature-based model has an important clinical value in the prognosis of ccRCC.

Consequently, we identified differentially expressed lncRNAs from public databases, and constructed a risk signature of ubiquitination-related lncRNAs and a nomogram, integrating the signature with clinical variables. Finally, a prognosis evaluation of the efficacy of this nomogram was carried out.

## Methods

### Data collection and processing

All transcriptome sequencing data and patients clinical information were downloaded from The Cancer Genome Atlas (TCGA) dataset, before 1st February 2021 (https://portal.gdc.cancer.gov/), and the protein ubiquitination regulatory pathway gene set “REACTOME_PROTEIN_UBIQUITINATION” (GSEA number: M27742) was recovered from the gene set enrichment analysis (GSEA) (https://www.gsea-msigdb.org/gsea/). The basic characteristic of included tumor tissues was presented in Table 1.

**Table 1:** Basic characteristics of included tumor specimens

Clinical variables	***N***
Case number	537
Age (years; mean ± SD)	60.59 ± 12.15
Gender (male/female)	346/171
Grade	
Gx	5
G1	14
G2	230
G3	207
G4	78
Unknown	3
Stage	
I	269
II	57
III	125
IV	83
Unknown	3
*T*	
T1	275
T2	69
T3	182
T4	11
*M*	
M0	426
M1	79
Mx	30
Unknown	2
*N*	
N0	240
N1	17
Nx	280

*SD* Standard deviation

### Acquisition of ubiquitination-related lncRNAs

One gene set (REACTOME_PROTEIN_UBIQUITINATION; M27742) including mRNAs related to ubiquitination of protein was derived from GSEA official websites to detect the ubiquitination-related lncRNAs. Protein-coding RNAs and lncRNAs data were extracted from the transcriptome profiling, and then mRNAs that regulate ubiquitination were selected through the GSEA database. The R (v. 3.6.1) Limma package was used to perform co-expression analysis of mRNAs regulating protein ubiquitination and lncRNAs (corfilter = 0.4, *p-*value = 0.001) in ccRCC patients. Long noncoding RNAs related to protein ubiquitination were extracted.

### Gene set functional enrichment analyses

Gene set functional enrichment analyses (GSEA) were used to evaluate lncRNAs biological functions in Gene Ontology (GO) and Kyoto Encyclopedia of Genes and Genomes (KEGG) databases. Functional enrichment with a *p*-value < 0.05 and a false discovery rate (FDR) of *q* < 0.25 were considered significant.

### Construction of clinical-prognostic index based on ubiquitination-related lncRNAs

Ubiquitination-related lncRNAs expression profiles were normalized by [log_2_(count+1)] transformation. The R survival package was used to perform a univariate Cox regression analysis of survival status and time using lncRNAs related to protein ubiquitination. A multivariate Cox regression analysis was applied to eliminate genes that failed to become independent indicators, and the remaining lncRNAs were used to establish a clinical-prognostic index with the best Akaike information criterion (AIC) value. The prognostic index (PI) formula was based on a linear combination of the relative expression level of lncRNAs multiplied regression coefficients (coef), which represented the relative weight of lncRNAs in the multivariate Cox regression analysis. This formula distinguished all ccRCC patients into a low-risk group and a high-risk group.

### Evaluation of clinical index diagnostic value

The Survminer package in the R was used to plot the survival curve, while the R pheatmap package was applied to draw the risk score scatter diagram, survival state plot, and risk heatmap, which indicated the diagnostic efficacy of this clinical-prognostic index. To determine if the PI was an individualized clinical-prognostic model, we perform a multivariate Cox regression analysis of the patients’ risk score, and their age, gender, pathological stage, historical grade, and TNM status to validate the independence of this PI. The survival ROC package was applied to calculate the Area Under Curve (AUC) of this PI and visualize the Receiver Operating Characteristic (ROC) curve. Clinical correlation analysis uses the ggpubr package for visual processing.

### Statistical analysis

All statistical analyses and visualizations of results were conducted using R software (3.6.1, http://www.r-project.org/) and strawberry Perl language (v5.30.1, http://www.perl.org/), PCA analysis was performed in the LIMMA package, and PCA visualization was performed using the scatterplot3d package. A *p*-value < 0.05 was considered in statistical analysis.

## Results

### Data acquisition

**Fig. 1:**
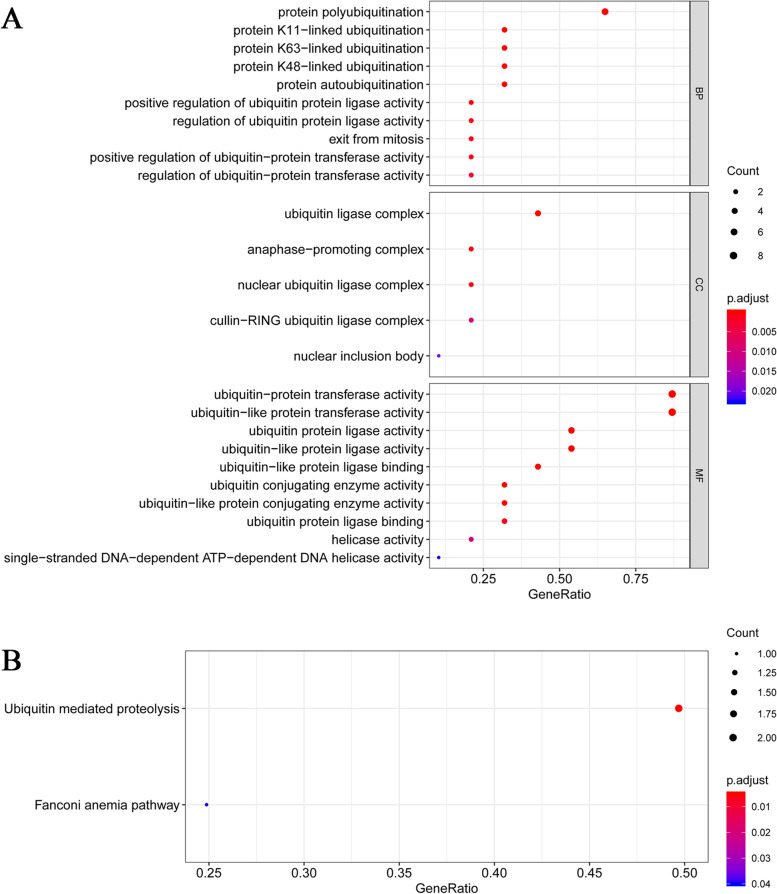
Gene set enrichment analysis of lncRNA signature by GO (**A**) and KEGG (**B**)

A total of 539 tumor samples collected from 530 ccRCC patients and 72 healthy kidney samples with gene expression data and clinical information — including age, gender, survival time, and TNM stage — were obtained from the TCGA database. Expression data of lncRNAs and mRNAs were also extracted from this database. The outcome of co-expression analysis between mRNAs regulating the ubiquitination of ccRCC and lncRNAs is available in Supplementary material 1. Based on co-expression analysis, 29 ubiquitination-related lncRNAs were identified to be significantly related to nine mRNAs regulating the ubiquitination of ccRCC (*p* < 0.05; Supplementary Fig. 1). To better understand the relationship between 29 ubiquitination-related lncRNAs and the ubiquitination of ccRCC, GSEA analysis was performed, and results are shown in Fig. 1. For the GO database, ubiquitination-related process and pathways, such as protein polyubiquitination, protein K11/63/48-related ubiquitination, and protein autoubiquitination, were significantly associated with 29 lncRNAs (Fig. 1A), while for the KEGG database, ubiquitin-mediated proteolysis was observed to be significantly enriched (Fig. 1B), indicating the high correlation of 29 lncRNAs with ubiquitination.

### Construction and evaluation of the risk signature model

**Fig. 2:**
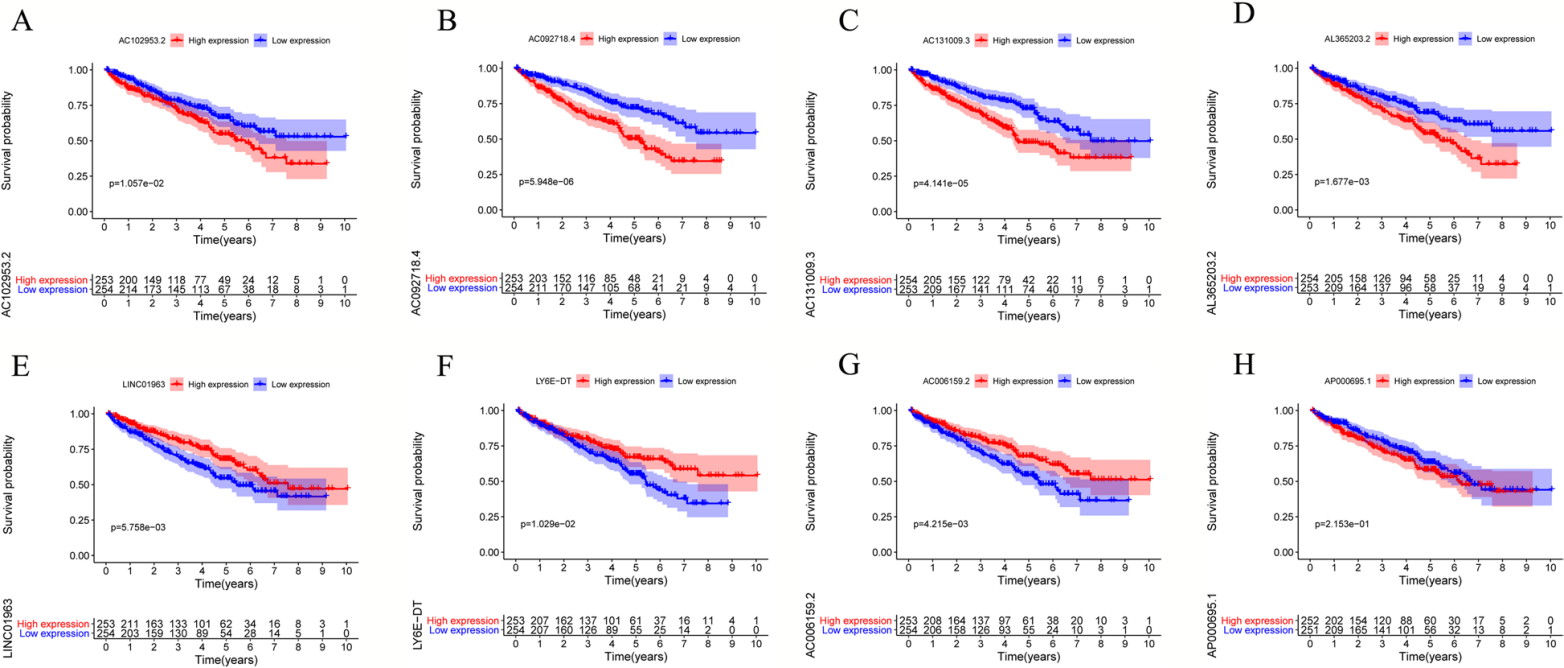
Survival analysis of 8 ubiquitination-related lncRNAs on the ccRCC patients, including AC131009.3 (**A**), AL365203.2 (**B**), AC102953.2 (**C**), LINC01963 (**D**), LY6E-DT (**E**), AC006159.2 (**F**), AP000695.1 (**H**), and AC092718.4 (**G**)

Univariate Cox regression analysis of 29 ubiquitination-related lncRNAs in 507 tumor specimens identified 14 lncRNAs to be significantly associated with the prognostic status of ccRCC (Table 2 and Supplementary Fig. 2), of which 8 lncRNAs (LINC01963, AC006159.2, LY6E-DT, AP000695.1, AC092718.4, AC131009.3, AL365203.2, and AC102953.2) were reported to have the most significant prognostic value (Table 2). Furthermore, specimens from the training set were classified into high-expression and low-expression groups according to the expression of each lncRNA. The survival analysis showed that 7 of 8 lncRNAs (LINC01963, AC006159.2, LY6E-DT, AC092718.4, AC131009.3, AL365203.2, and AC102953.2) can independently predict the survival status of ccRCC patients (*p* < 0.05; Fig. 2). Additionally, we compared the expression of each lncRNA with clinical variables extracted from the TCGA database, including age, gender, tumor grade, tumor stage, and TNM stage. As shown in Fig. 4, all 8 lncRNAs were found to be associated with tumor severity, tumor stage, and TNM stage in varying degrees *(p <* 0.05; Supplementary Fig. 3). These results showed that 8 lncRNAs were significantly correlated with the prognosis value of ccRCC patients.

**Table 2:** Expression and Cox regression analysis of the prognostic ubiquitination-regulation-associated lncRNAs in ccRCC patients

LncRNA	Expression	Univariate COX regression analysis			Multivariate COX regression analysis			
	Mean ± SD	***HR***	95% ***CI***	***p***-value	***HR***	Coefficient	95% ***CI***	***p***-value
LINC01963	2.03 ± 0.51	0.56	0.40–0.78	< 0.001	0.61	−0.50	0.43–0.85	0.0033
AC006159.2	0.50 ± 0.50	0.56	0.3–-0.82	0.0025	0.74	−030	0.51–1.07	0.11
LY6E-DT	0.89 ± 0.65	0.66	0.51–0.85	0.0014	0.81	−0.21	0.62–1.05	0.10
AP000695.1	1.173 ± 0.69	1.50	1.20–1.86	< 0.001	1.26	0.23	0.98–1.61	0.070
AC092718.4	2.02 ± 0.66	1.71	1.40–2.09	< 0.001	1.27	0.24	1.00–1.60	0.050
AC131009.3	1.07 ± 0.50	2.05	1.58–2.65	< 0.001	1.58	0.46	1.14–2.19	0.0055
AL365203.2	1.54 ± 0.48	2.33	1.72–3.16	< 0.001	1.59	0.46	1.12–2.25	0.010
AC102953.2	0.70 ± 0.34	2.64	1.71–4.07	< 0.001	1.70	0.53	1.06–2.70	0.026
AC005083.1	2.96 ± 0.95	0.77	0.65–0.91	0.0023				
AC005261.1	2.78 ± 0.55	1.83	1.38–2.42	< 0.001				
AC093673.1	3.41 ± 0.63	1.80	1.41–2.29	< 0.001				
AL136295.7	1.65 ± 0.52	1.63	1.21–2.19	0.0013				
NORAD	6.06 ± 0.44	0.54	0.37–0.78	0.0012				
RUSC1-AS1	1.17 ± 0.63	1.61	1.27–2.05	< 0.001

**Fig. 3:**
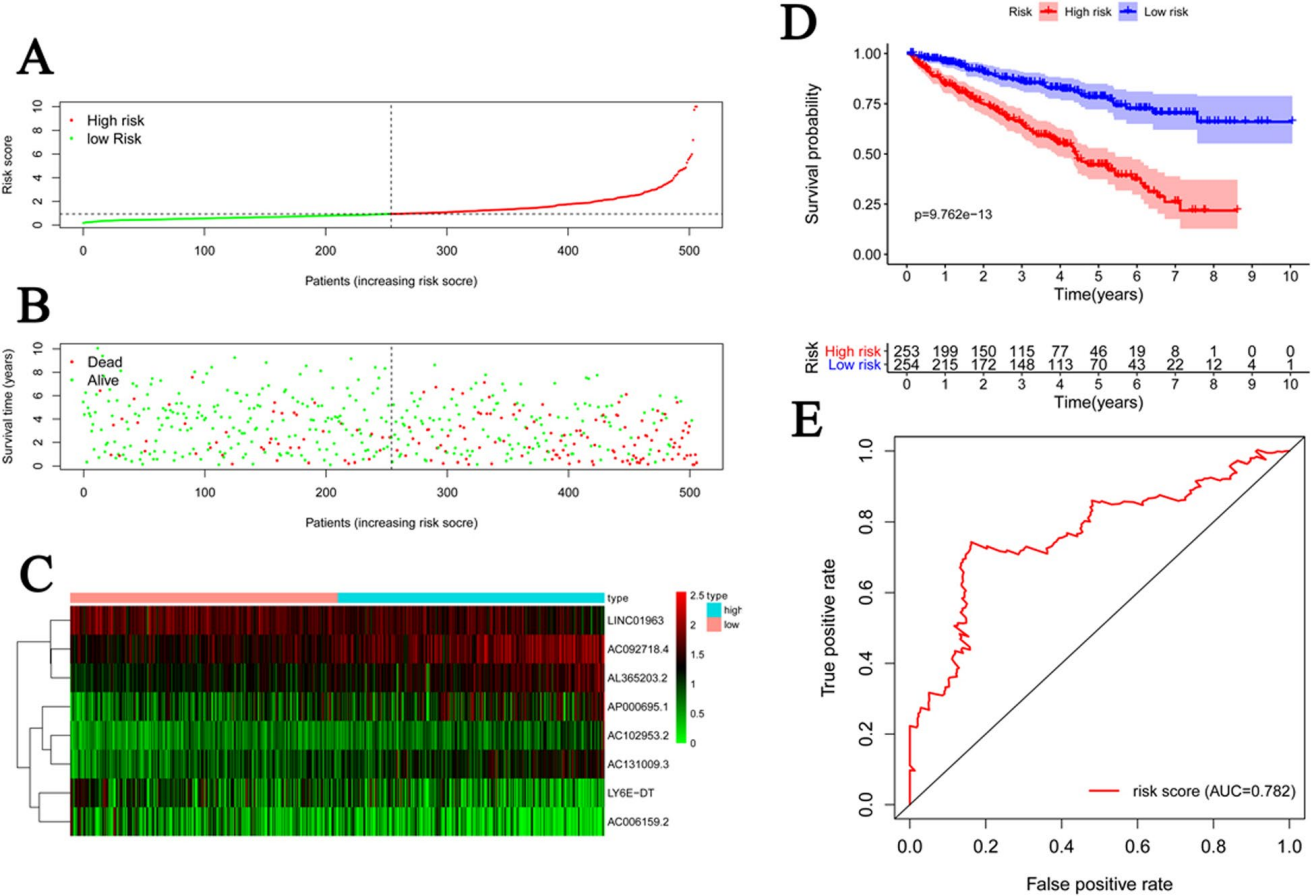
Analysis of risk signatures on the prediction of survival stautus. **A** risk score distribution, **B** ccRCC pateints’ survivial status, **C** gene expression in high-risk and low-risk groups, **D** Kaplan-Meier curves of risk signature, and **E** ROC curves in ccRCC patients

Next, the predictive model was defined as a linear combination of the gene expression levels of 8 lncRNAs, whose relative coefficient weights were derived from the multivariable Cox regression (Table 2). The risk score was calculated based on the sum of each lncRNA coefficient and each specimen’s corresponding expression. Then we divided these specimens in the training set into high-risk and low-risk groups according to the median risk score of 0.93 (Fig. 3A–C) and performed the survival analysis between the two groups (Fig. 3D–E). Patients in the low-risk group tended to have a longer OS compared to the high-risk group (*p* < 0.001; Fig. 3D). Moreover, the predicting ability of the 8-lncRNA signature model was assessed by the ROC curve, and the overall AUC of 0.782 (95% CIs) predicting overall survival indicated a relatively good performance on ccRCC diagnostic value (Fig. 3E).

**Fig. 4:**
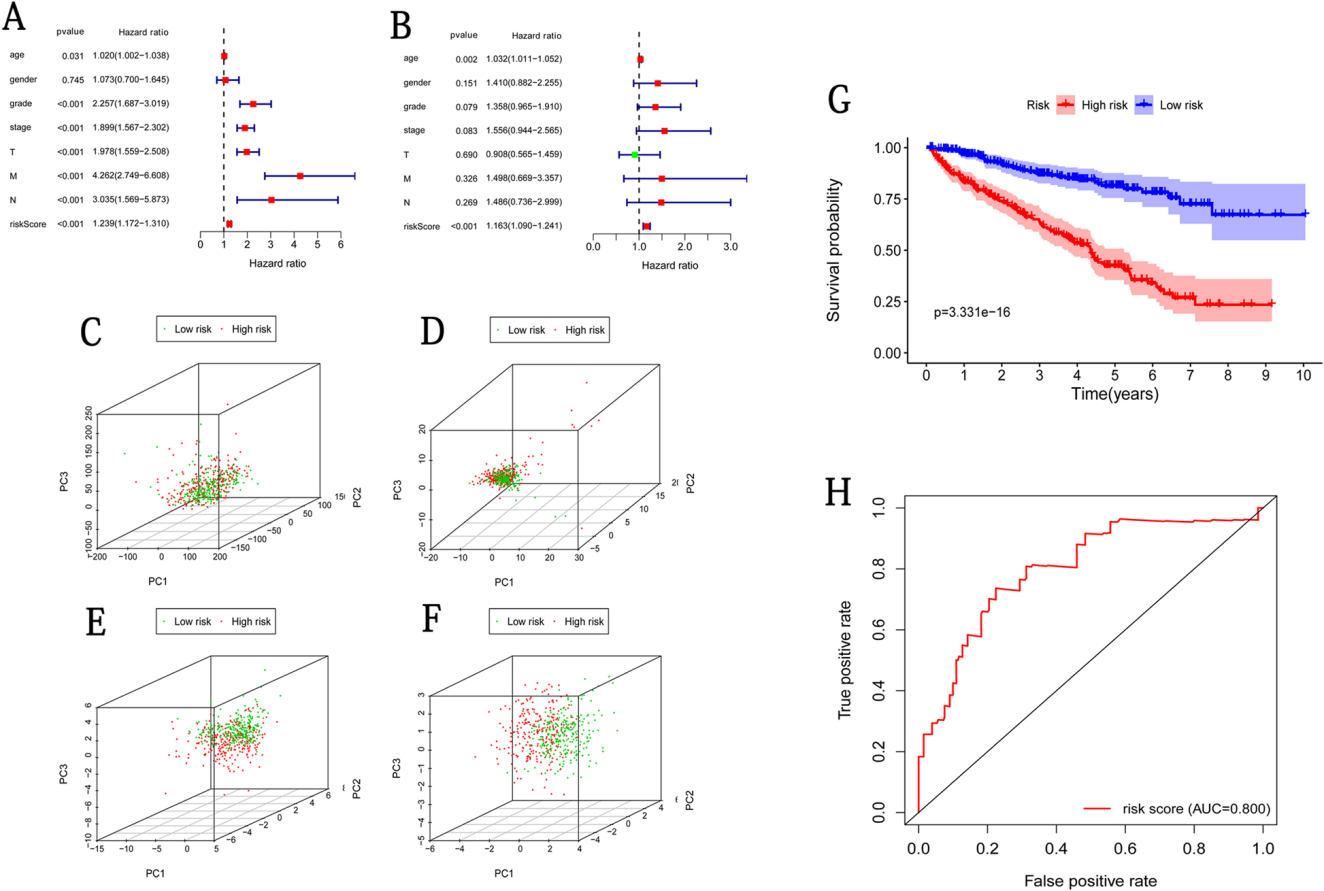
Prognositic efficacy of 8-lncRNA-based model on the prognosis of ccRCC. Prognostic value of clinicopathological characteristics and 8-lncRNA RiskScore by univariate (**A**) and multivariate (**B**) Cox regression analysis. Principal component analysis (PCA) based on total mRNA (**C**), ubiquitination-related mRNAs (**D**), ubiquitination-related lncRNAs (**E**), and 8 ubiquitination-related risk signatures (**F**). Analysis of ubiquitination-associated clinical-prognostic index (UCPI) on the prediction of survival stautus. **G** Kaplan-Meier curves of UCPI and **H** ROC curves in ccRCC patients

Finally, we evaluated the prognostic value of clinicopathological characteristics and 8-lncRNA RiskScore by univariate and multivariate Cox regression analysis. The univariate Cox regression analysis (Fig. 4A) reported six significant risk factors for ccRCC patients OS, including tumor grade (*HR*, 2.26), stage (*HR*, 1.90), TNM stage (HR of *T*, *N*, *M*: 1.98, 4.26, and 3.04, respectively) and RiskScore (*HR*, 1.24), whereas only age and RiskScore were identified as independent risk factors for ccRCC patients OS by multivariate Cox regression analysis (HR of age, 1.03; HR of RiskScore, 1.16; Fig. 4B). Principal component analysis of the low-risk and high-risk groups showed that, compared with total mRNA, ubiquitination-related mRNAs, and ubiquitination-related lncRNAs (Fig. 4C–E), the 8-lncRNA model could significantly separate the specimens into the two groups (Fig. 4F), suggesting the model high predicting value on the survival status of ccRCC.

### Identification of 8 lncRNAs clinical characteristics

Multivariable Cox regression analysis on clinical characteristics and lncRNAs identified that age and risk score were independent risk factors for ccRCC patients OS (Fig. 4B). A ubiquitination-associated clinical-prognostic index (UCPI) was established in a combination of age and 8-lncRNA model, based on coefficients derived from the multivariable Cox regression analysis. Moreover, UCPI prognostic efficacy on ccRCC patients’ OS was evaluated by Kaplan-Meier curves, and the results showed that the overall OS in the low-risk group was significantly higher than those in the high-risk group (*p* < 0.0001; Fig. 4G). The ROC curve reported that the AUC for the overall ccRCC patients’ OS rate was 0.800, which was higher than the AUC of 0.782 using the 8-lncRNA model (Fig. 4H). These results demonstrated that UCPI prediction performance was good for ccRCC patients’ prognosis.

## Discussion

We systematically collected data from the TCGA dataset and extracted ubiquitination-related lncRNAs. Then, a risk signature model containing 8 ubiquitination-related lncRNAs was established through Cox regression analysis. Finally, a nomogram was built based on age and risk score, and the prediction value was good in the ccRCC cohort set.

Eight ubiquitination-related lncRNAs were included in this UCPI, including LINC01963, AC006159.2, LY6E-DT, AP000695.1, AC092718.4, AC131009.3, AL365203.2, and AC102953.2. A recent study designed to identify and establish the immune risk signature for lung adenocarcinoma (LUAD) prognosis reported a seven immune-related lncRNA model containing AP000695.1 [19]. Combined with our results, AP000695.1 may be closely related to tumor prognosis, including LUAD and ccRCC, whereas its expression in other tumors can be further explored. Besides, AL365203.2 was also detected and included in a prognosis-predictive signature and nomogram for hepatocellular carcinoma (HCC) [20]. In this study, a set of lncRNA based on autophagy function was identified by co-expression analysis and showed good reliability and accuracy to predict 1-year and 3-year OS of HCC patients. Considering the essential link between ubiquitin signaling and autophagy, through promoting autophagy-dependent degradation by a variety of ubiquitin chains, AL365203.2 was included in both ubiquitination-related UCPI and autophagy-related nomogram for HCC, implying its potential mechanisms in tumor progression and metastasis. Importantly, lncRNA LINC01963 may play a crucial role in various carcinomas. A study based on 67 pancreatic cancer patients investigated the expression of LINC01963, both in vivo and in vitro [21]. The lncRNA LINC01963 has been confirmed to be significantly lower in pancreatic carcinoma tissues and cell lines by targeting miR-641/TMEFF2, whereas silence of LINC01963 could improve the development of cell culture tumors. For oral and oropharyngeal squamous cell carcinoma (OSCC/OPSCC), a strong correlation between LINC01963 and OSCC/OPSCC prognosis has been observed by an autophagy-related prediction model containing nine lncRNAs [22]. Consistent with our study, the outcomes in these studies supported the multiple functions of LINC01963 in a variety of carcinomas, including pancreatic carcinoma, ccRCC, and OSCC/OPSCC. Nevertheless, no study was conducted to explore other ubiquitination-related lncRNAs in the proposed UCPI.

Ubiquitination and its biological processes were significantly correlated with the VHL-hypoxia-inducible factors (HIFs) axis — well-characterized and implicated as connections to the pathogenesis of ccRCC [23]. The ubiquitination factor E4B (UBE4B) has been extensively explored in ccRCC tumor tissues and cell lines and may act as an oncogene in ccRCC development, apoptosis, and cell proliferation regulation [24]. Furthermore, degradation and ubiquitination of ARHGEF7, mediated by KLHL2, was observed in ccRCC cell cultures, suggesting the essential role of ubiquitination in the development of ccRCC [25, 26]. Likely, ubiquitin-like PHD and RING finger domain 1 (UHRF1) has been reported to promote non-degradative ubiquitination of p53, suppress p53 pathway activation, and p53-dependent apoptosis in ccRCC cells [27, 28]. Taken together, ubiquitination seems to play a pivotal role in ccRCC pathogenesis and prognosis, supporting the good performance of UCPI derived in this study for ccRCC.

In this study, we constructed one UCPI based on ubiquitination function and clinical variables, which performed well in survival outcome prediction of ccRCC patients. Previous studies have been performed to explore biomarkers for ccRCC prognosis. Recently, similar methods have been used, and a brand new autophagy-related seven-gene prognostic risk signature was reported [29]. However, this risk signature included the differential expressed genes (DEGs) as well as autophagy-related genes (ARGs) for regression analysis, and some ARGs did not reach DEGs criteria, which may contribute to the potential bias for this risk signature. Additionally, ubiquitination was considered to control multiple steps of autophagy, essential for autophagy flux regulation in a variety of signaling pathways [30–32]. DEGs related to ubiquitination may provide a superior prediction value than those related to ARGs. Interestingly, Siteng Chen et al. [33] developed a machine learning histopathological image signature to predict ccRCC diagnosis and survival. This image signature could accurately distinguish ccRCC from other cancer pathological types with an average AUC of approximately 90%. Moreover, they also constructed an integrated nomogram based on their computational recognition risk score and clinicopathological factors, whereas the long-term performance of this nomogram based on machine learning still needs to be further validated. Small nucleolar RNAs (snoRNAs) were demonstrated to play significant roles in tumorigenesis and may exhibit prognostic value. In a recent study, a six-snoRNA signature was identified as an independent ccRCC diagnosis and prognosis indicator, and the Wnt signaling pathway was suggested to be crucial in ccRCC pathogenesis [34]. Considering that snoRNAs are a kind of noncoding RNAs guiding site-specific posttranslational modification, the effect and mechanisms of snoRNAs remained unclear in ccRCC, which limited the clinical practice of this risk signature. Previous review has pointed out that poor level of evidence during the routine use was noticed for multiple prognostic models and nomograms, while molecular markers derived from various orgins and functions should be taken into consideration [35].

There were some limitations in our study. First, this UCPI was established from the TCGA database instead of ccRCC tumor specimens, which should be validated in a prospective and long-term cohort in clinical practice. Then, we considered the confounding factors of clinical variables on ccRCC prognosis and constructed the nomogram containing the significant clinical factors. The inherent bias and unknown confounders may contribute to the limitations. Last but not least, only ccRCC tumor issues were included for the dectection and validation of ubiquitination-related RNAs, while the serological samples were ignored. We belived that biomarkers derived from serological samples should contribute to the construction of UCPI, and further exploration should be carried out.

## Conclusions

Finally, we developed and validated an eight-lncRNA UCPI for ccRCC patients’ prognosis. This UCPI performed well in the prediction of ccRCC patients’ survival status with an AUC of 0.800, providing a novel insight for ccRCC patients’ prognosis and a guide for further individualized therapy. Further extensive study should be designed to prospectively validate the prognostic UCPI model in a large-scale cohort. Moreover, our ubiquitination-related model strongly suggested the crucial effect of ubiquitination on the progression of renal carcinoma.

## Supplementary Information

**Additional file 1: Supplementary Fig. 1.** Coexpression analysis of 9 ubiquitination-related mRNAs and 29 lncRNAs.

**Additional file 2: Supplementary Fig. 2.** Univariate Cox regression analysis of 29 ubiquitination-related lncRNAs in 507 tumor specimens identified 14 lncRNAs to be significantly associated with the prognostic status of ccRCC.

**Additional file 3: Supplementary Fig. 3.** Expression analysis of 8 ubiquitination-related lncRNAs on clinical characteristics, including age (**A**), gender (**B**), tumor grade (**C**), tumor stage (**D**), tumor TNM stage (**E**-**G**).

## Data Availability

The datasets analyzed were acquired from The Cancer Genome Atlas (TCGA) database (https://portal.gdc.cancer.gov/) and GEO database (http://www.ncbi.nlm.nih.gov/geo/).
