# Supplementary figures and images for: Construction of an individualized clinical prognostic index based on ubiquitination-associated lncRNA in clear cell renal cell carcinoma patients
Source: World J Surg Oncol. 2022 May 10;20:148. doi: 10.1186/s12957-022-02618-x (PMC9087998; doi:10.1186/s12957-022-02618-x)

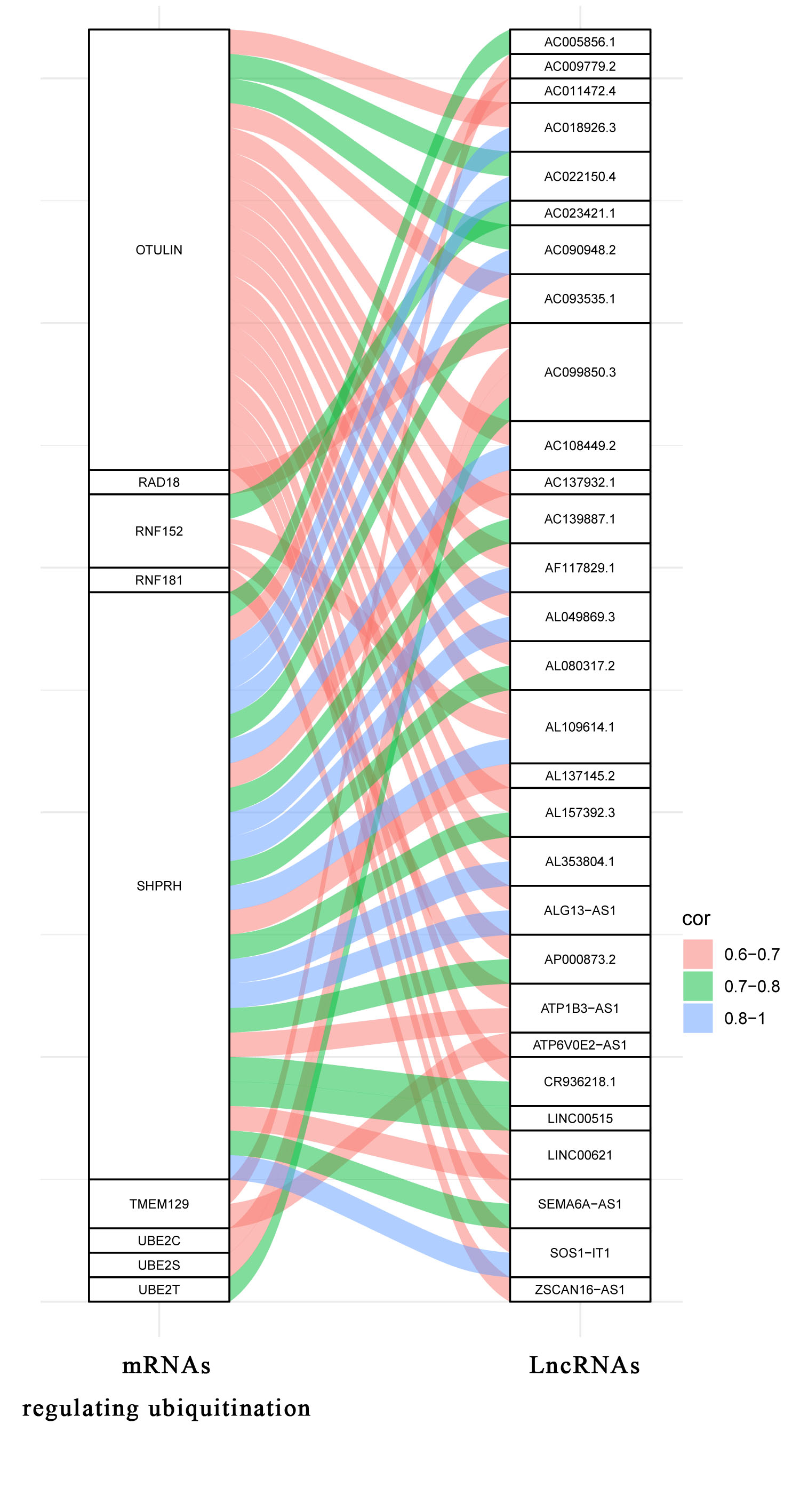

Supplement: Supplementary file 1 — Additional file 1: Supplementary Fig. 1. Coexpression analysis of 9 ubiquitination-related mRNAs and 29 lncRNAs. [file 12957_2022_2618_MOESM1_ESM.jpg]

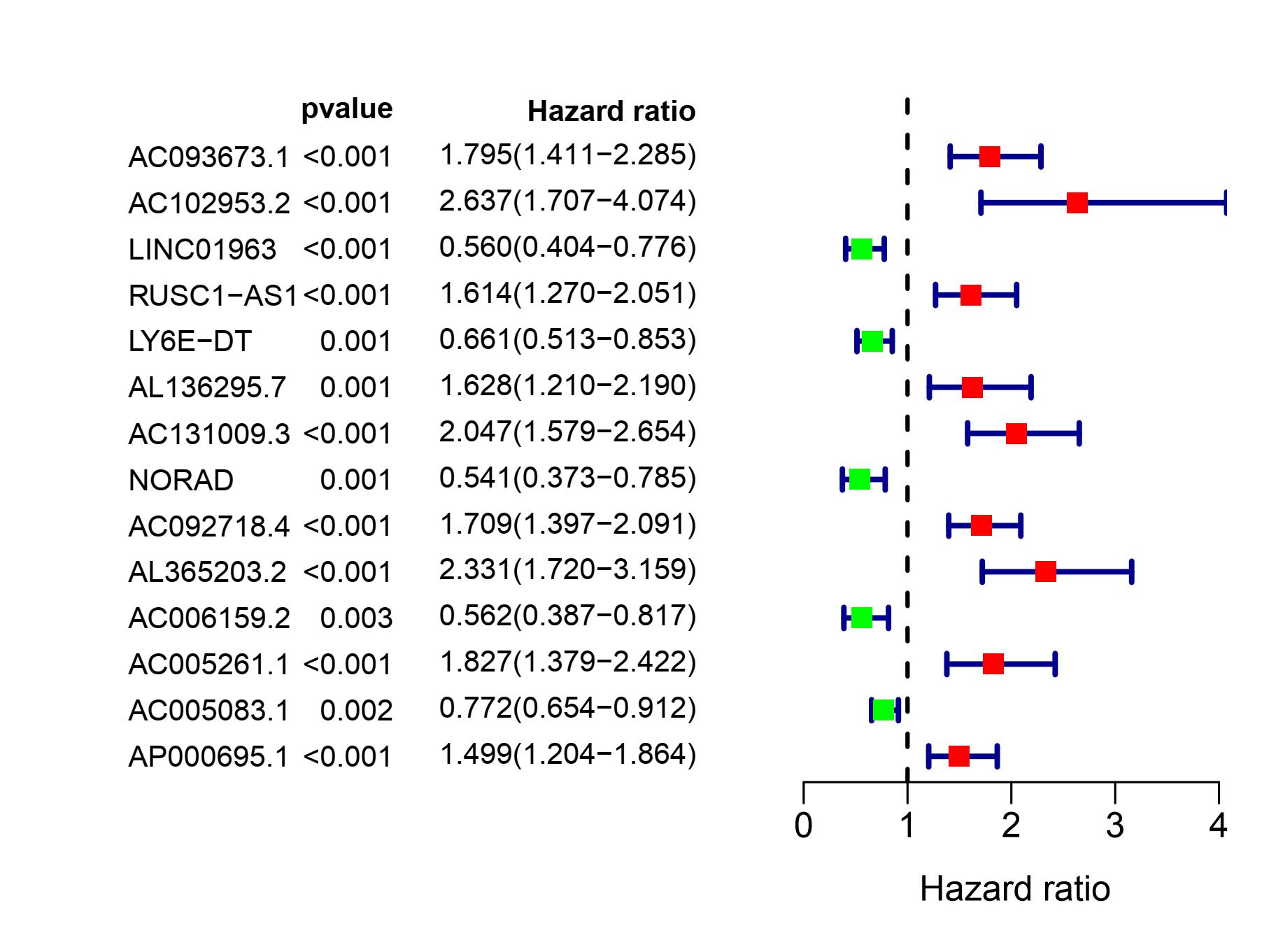

Supplement: Supplementary file 2 — Additional file 2: Supplementary Fig. 2. Univariate Cox regression analysis of 29 ubiquitination-related lncRNAs in 507 tumor specimens identified 14 lncRNAs to be significantly associated with the prognostic status of ccRCC. [file 12957_2022_2618_MOESM2_ESM.jpg]

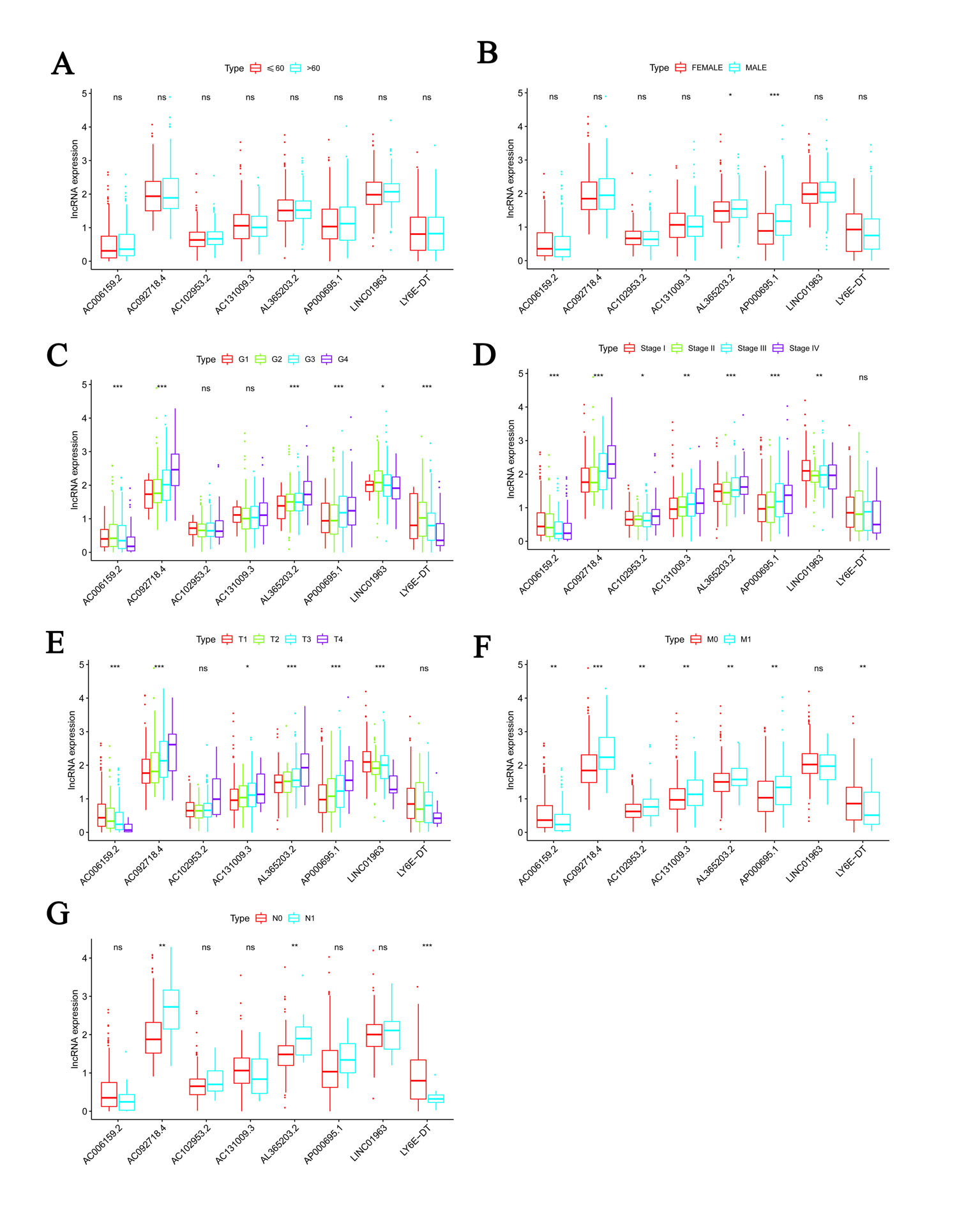

Supplement: Supplementary file 3 — Additional file 3: Supplementary Fig. 3. Expression analysis of 8 ubiquitination-related lncRNAs on clinical characteristics, including age (A), gender (B), tumor grade (C), tumor stage (D), tumor TNM stage (E-G). [file 12957_2022_2618_MOESM3_ESM.jpg]
